# List of Diseases at the Bath City Dispensary, from December 31, 1801, to January 30, 1802

**Published:** 1802-04

**Authors:** W. White

**Affiliations:** Bath


*List of Diseases at the Bath City Dispensary, from December* 31, 1801, *to January* 30, 1802.

| | | | |
|---|---|---|---|
| Angina - - - - - - - | 1 | Peripneumonia Notha - - | 2 |
| Abſceſs - - - - - - - | 2 | Pulmonary Complaints - - | 10 |
| Anthrax - - - - - - - | 1 | Phthiſis Pulmonalis - - - | 4 |
| Aſthma - - - - - - - | 7 | ——— Incipiens - - - | 1 |
| Aſcites - - - - - - - - | 1 | Pertuſſis - - - - - - - | 3 |
| Anaſarca - - - - - - - | 2 | Phyſconia - - - - - - | 1 |
| Bilious Complaints - - - | 3 | Prolapſus Uteri - - - - - | 1 |
| Bloody Urine - - - - - | 2 | Pſora - - - - - - - - - | 1 |
| Catarrh - - - - - - - - | 14 | Rheumatiſmus Acutus - - | 11 |
| Contuſion - - - - - - | 1 | Synochus Bilioſa - - - | 10 |
| Carcinoma - - - - - - | 1 | ——— Pleuritica - - | 2 |
| Chloroſis - - - - - - - | 4 | ——— Enteritica - - | 1 |
| Diarrhœa - - - - - - | 5 | ——— Dyſenterica - - | 1 |
| Eryſipelas - - - - - - | 3 | ——— Cholerica - - | 1 |
| Eruptiones - - - - - - | 3 | Scarlatina - - - - - - - | 1 |
| Fever - - - - - - - - | 8 | Suppuration - - - - - - | 1 |
| Gaſtrodynia - - - - - - | 3 | Syphilis - - - - - - - | 3 |
| Gonorrhœa - - - - - - | 1 | Sciatica - - - - - - - - | 1 |
| Hæmoptyſis - - - - - - | 2 | Scrophula - - - - - - - | 3 |
| Hæmorrhagia (inteſtinal) - | 1 | Strain - - - - - - - - - | 3 |
| Hæmorrhois - - - - - - | 2 | Typhus Gravior - - - - | 4 |
| Hernia Humoralis - - - | 2 | Tertian - - - - - - - | 1 |
| Hernia - - - - - - - - | 1 | Tuſſis - - - - - - - - | 7 |
| Herpes - - - - - - - - | 1 | Tumor Mammæ - - - - - | 1 |
| Icterus - - - - - - - - | 2 | Urticaria - - - - - - - | 1 |
| Lepra - - - - - - - - | 1 | Ulcerated Legs - - - - | 7 |
| Menorrhagia - - - - - - | 1 | Variola - - - - - - - | 4 |
| Ophthalmia - - - - - - | 1 | | |
| | | Total | 161 |

*Bath, Feb.* 7, 1802.

W. WHITE.

ACCOUNT

